# Prognostic value of radical cystoprostatectomy in men with bladder cancer infiltrating prostate versus co-existing prostate cancer: a research study

**DOI:** 10.1186/1471-2490-10-16

**Published:** 2010-09-22

**Authors:** Piotr Bryniarski, Mieczysław Fryczkowski, Paweł Pawlaczek, Krzysztof Pliszek, Grzegorz Prokopowicz, Zbigniew Kaletka, Andrzej Paradysz

**Affiliations:** 1 Medical University of Silesia, Department of Urology, 3 Maja street, Zabrze 41-800, Poland; 2 Provincial Hospital, Armii Krajowej street 101, Bielsko-Biala 43-316, Poland

## Abstract

**Background:**

The aim of the following study is to evaluate the advancement of incidentally diagnosed prostate cancer in specimen after cystoprostatectomies caused by muscle-invasive bladder cancer. Secondly we assessed the survival in patients after radical cystoprostatectomy whose postoperative specimen was characterized by the presence of co-existing prostate cancer or prostate infiltration by urothelial bladder cancer.

**Methods:**

Between 1993 and 2009 a total of 320 patients with muscle-invasive bladder cancer underwent cystoprostatectomy. The first analyzed group consisted of 52 patients with bladder cancer infiltrating prostate, while the second group consisted of 21 patients with co-existing prostate cancer. In all patients cancer specific survival and progression were analyzed. Average follow up was 75.2 months (range: 0 - 181).

**Results:**

Cancer-specific survival was significantly shorter in group I (p = 0.03). Neoplastic progression in patients from group I was observed in 42.2% of patients, while in patients from group II in 23.6% of patients (p = 0.04). No statistical difference was observed in the percentage of positive lymph nodes between the groups (p = 0.22). The median Gleason score in patients with co-existing prostate cancer was equal to 5. The stage of prostate cancer pT_2_/pT_3 _was equal to 20 (96%)/1 (4%) patients. 12 (57%) prostate cancers were clinically insignificant. Biochemical recurrence occurred in 2 (9%) patients.

**Conclusions:**

1. Incidentally diagnosed prostate cancer in specimen after cystoprostatectomies is frequently clinically insignificant and characterized by low progression.

2. Patients with bladder cancer infiltrating prostate are characterized by higher percentage of progression and death in comparison with patients with co-existing prostate cancer.

## Background

Cystoprostatectomy is a treatment of choice in men suffering from muscle-invasive bladder cancer. However, this treatment involves the risk of complications like incontinence, sexual and metabolic disorders [1].

Complete histopathologic examination not only enables evaluating the stage and grade of the bladder cancer, but also the prostate. This is extremely important because in autopsy examinations in 30% of men up to the age of 50, single cancer cells are found in the prostate. The percentage in 80-year-old men becomes higher, up to 70% [2].

Incidental prostate cancers of that type are usually clinically insignificant, quite well differentiated, not exceeding the border of the prostate capsule and diagnosed in 5 - 36% of cases [3,4]. The frequency of urothelial cancer infiltration of the prostate is equal to 5 - 40% [5].

In the majority of operated patients the intraoperative advancement of the bladder cancer is higher than it was supposed during preoperative examinations. Most often the histopathologic diagnosis either:

1. confirms the urothelial cancer infiltration of the prostate and even urethra, or

2. confirms the diagnosis of independently co-existing prostate cancer.

Both situations are unfavorable prognostic factors for the operated patient. A high percentage of complications after cystoprostatectomy forced the urologists to search for the new sparing operative techniques, namely, cystectomies sparing the prostate capsule, its apex or the whole prostate gland [1,6,7]. Nevertheless such treatment carries the risk of non-radical cancer removal [8].

In the situation where removal of the bladder without removing the prostate is becoming more and more fashionable and frequently utilized, a question concerning the clinical significance of the abovementioned changes arises. The aim of the following study is to evaluate the advancement of incidentally diagnosed prostate cancer in specimen after cystoprostatectomies caused by muscle-invasive bladder cancer. Another aim of the research was to evaluate the survival in patients after radical cystoprostatectomy whose postoperative specimen was characterized by the presence of co-existing prostate cancer or infiltration of the prostate by urothelial cancer.

## Methods

The study has been approved by the Silesian Medical University Ethics Committee and has therefore been performed in accordance with the ethical standards laid down in the 1964 Declaration of Helsinki. All patients gave their informed consent prior to their inclusion in the study.

In the period of time between December 1993 and March 2009 in the Department of Urology of the Medical University in Zabrze and the Urologic Department of the Provincial Hospital in Bielsko-Biała 320 patients with bladder cancer underwent cystoprostatectomy. 77 patients were subject of the current analysis. The patients were divided into two groups. The first one consisted of 52 patients with bladder cancer infiltrating prostate, while the second group consisted of 21 patients with co-existing prostate cancer. 4 patients were diagnosed with both co-existing prostate cancer and bladder cancer infiltrating prostate. These 4 patients were excluded from the research in the evaluation of survival. 21 patients from the second group accounted for 6.5% of all men with bladder cancer treated in the evaluated period of time in both centers.

None of the patients was subject to neoadjuvant therapy. In 14 (18.1%) patients orthotopic intestinal bladder was made, in 29 (37.6%) ureterocutaneostomies and in 34 (44.1%) ileal conduit. None of the analyzed patients died during the operation. According to the 2002 TNM classification the infiltration of prostate by urothelial bladder cancer means stage pT4a. Co-existing prostate cancer was considered insignificant if all of the features were observed: preoperative PSA < 10 ng/ml, Gleason score < 7, cancer lesion less than 0,5 ml and stage pT_2 _(organ-confined). Whole-mount sectioning of the prostate was conducted by 2 pathologists.

Patients after the surgery were monitored every 3 months during first year, for another 2 years - every 6 months and once a year 3 years after the surgery. Control tests included creatinine concentration, electrolytes, PSA concentration, urine stasis in the upper urinary tract (ultrasonography), chest radiography and pelvic/abdominal CT once a year.

In all patients the following criteria were analyzed: age, cancer specific survival, the frequency of complications and deaths, local recurrences, positive surgical margins, distant metastases, adjuvant chemotherapy, number of lymph nodes dissected and creatinine concentration before and after the surgery. Biochemical recurrence of prostate cancer was diagnosed if two consecutive serum PSA tests exceeded the level of 0.2 ng/ml. Survival time was 0 - 181 months (on average: 75.2 months).

The statistical analysis was performed by means of Statistica Statsoft v. 8.0. For continuous variables U-Mann-Whitney test was used. For categorical variables the Chi-square test was applied. Kaplan-Meier curves were used for the evaluation of cancer-specific survival with log-rank test. Proportional hazard (Cox) regression model was used to assess the influence of stage (T), grade (G), positive nodes (N), positive surgical margin, adjuvant chemotherapy and coexisting prostate cancer or pT4a bladder cancer on cancer specific survival.

## Results

The average age of operated patients in group I was 62.7 years, while in group II 67.4 years. The difference was at the verge of statistical significance (p = 0.06).

**Figure 1:**
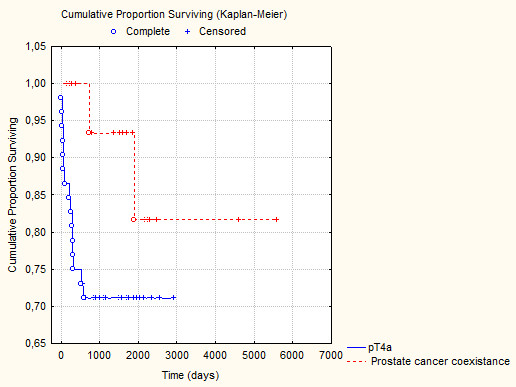
**Cancer specific survival (Kaplan-Meier curves) of the analyzed patients (log-rank test p = 0,04)**.

The stage was obviously significantly higher in group I (p < 0.001). However, no significant statistical difference was observed in the percentage of positive lymph nodes 49.9% vs. 47.5% respectively (p = 0.22) (see table 1). Survival time was significantly shorter in group I (p = 0.03, see figure 1). Deaths also occurred more frequently in group I at the verge of statistical significance (p = 0.07). Proportional hazard (Cox) regression showed statistically significant influence of stage (HR: 1.2; p = 0.04), positive nodes (HR: 1.4; p = 0,03), grade (HR: 1.1; p = 0.03), co-existing prostate cancer/pT4a bladder cancer (HR: 2.2; p = 0.04), adjuvant chemotherapy (HR: 1.1; p = 0.02) and positive surgical margin (HR: 1.2; p = 0,03) on cancer-specific survival.

**Table 1:** Comparison of patients with bladder cancer infiltrating prostate (group I) and co-existing with prostate cancer (group II).

		Group I (n = 52)	Group II (n = 21)	p value
**Age**		62.7	67.4	0.06

**pT**	1	0	3 (14,2%)	< 0.001
		
	2	0	7 (33.3%)	
		
	3	0	11 (52.3%)	
		
	4a	52 (100%)	0	

**N**	0	26 (50%)	11 (52.3%)	0.22
		
	1	2 (3.8%)	3 (14.2%)	
		
	2	24 (46.1%)	7 (33.3%)	

**G**	1	5 (9.6%)	4 (19%)	0.16
		
	2	20 (38.4%)	11 (52.3%)	
		
	3	27 (51.9%)	6 (28.5%)	

**Creatinine concentration before the surgery (mg/dl)**		1.31 (0.78-3.65)	1.35 (1.05-1.98)	0.69

In our study 50 patients (96,1%) had positive cystoscopy in terms of prostate infiltration prior to bladder removal.

Generally, neoplastic progression in patients from group I after 60.5 months of observation was equal to 42.2%, while in patients from group II after 83.6 months of observation - 23.6% (p = 0.04). However, no statistical significance concerning individually the percentage of distant metastases, complications after the surgery or local recurrences was observed. The average creatinine concentration in group I before the surgery was 1.31 mg/dl and 1.19 mg/dl after the surgery (p = 0.12). The average creatinine concentration in group II before the surgery was 1.35 mg/dl and 1.15 mg/dl after the surgery (p = 0.11).

In group II prostate cancer was characterized by high histopathologic differentiation and low clinical advancement. The median total Gleason score in patients with co-existing prostate cancer was equal to 5. The stage of prostate cancer pT_2_/pT_3 _was diagnosed in 20 (96%)/1 (4%) patients. 12 (57%) prostate cancers were clinically insignificant.

4 patients excluded from the research (with co-existing prostate cancer and prostate infiltration by urothelial bladder cancer) had cancer-specific survival comparable with group I (log rank; p = 0,8). In 2 (50%) of them prostate cancer was clinically insignificant. 2 (8%) patients had prostate cancer with score ≥7 in Gleason scale. Interestingly, both patients were also diagnosed with infiltration of prostate by urothelial bladder cancer.

After almost 7 years of postoperative observation in patients from group II PSA concentration ranged between 0.04 and 2.9 ng/ml (on average 0.25 ng/ml). Biochemical recurrence occurred in 2 (9%) patients, in both of them 5 years after the surgery (PSA concentration - 2.9 and 2.0 ng/ml respectively). Complications in the form of pyelonephritis in 5 patients, intestinal fistula - 1 patient, the narrowing of the anastomosis of the urethra and the bladder (1 patient), postoperative obstruction (1 patient) or bleedings (2 patients) occurred only in group I (see table 2).

**Table 2:** Postoperative characteristics of patients with bladder cancer infiltrating prostate (group I) and with co-existing prostate cancer (group II).

	Group I (n = 52)	Group II (n = 21)	p value
**Creatinine concentration after the surgery (mg/dl)**	1.19 (0.7-2.6)	1.15 (1-1.31)	0.91

**Positive surgical margin**	9 (17,3%)	0	0.04

**Mean number of lymph nodes dissected**	20,2	19,6	0,83

**Local relapse**	2 (3.8%)	1 (4.7%)	0.85

**Distant metastases after operation**	5 (9.6%)	2 (9.4%)	0.37

**Adjuvant chemotherapy**	26 (50%)	3 (14,2%)	0,04

**Postoperative complications**	5 (9.6%)	0	0.14

**Death (cancer-specific)**	15 (28.8%)	2 (9.5%)	0.07

**Follow-up (months)**	60.5 (0-76)	83.6 (9-181)	0.09

## Discussion

Cystoprostatectomy carries the risk of several functional complications like urinary incontinence and erection disorders.

The studies show that during the day incontinence occurs in 5 - 18% of patients, while at night in 13 - 27% of patients. Erectile dysfunction appears in 75 - 80% of patients [9]. In a situation when organ sparing surgeries, protecting the patient's basic vital functions, are becoming more and more fashionable and frequently utilized, a question concerning the oncological radicality of the abovementioned procedure arises. Close monitoring of such patients with regard to local recurrence and distant metastases is crucial, taking into account the fact that majority of local relapses occur within the first 2 years after the surgery. The relapse is diagnosed in about 10% of patients [10-12]. The frequency of distant metastases after organ sparing surgeries is estimated at 32 - 34% [10,13]. In the following research the frequency of local recurrences in patients with pT_4 _was 4.8%, while the frequency of distant metastases was equal to 9.6%. The frequency of prostate infiltration by urothelial cancer, as has already been stated in the introduction, is estimated at 5 - 40% [5]. It has to be stressed that primary urothelial cancer can very rarely develop in the prostatic part of the urethra. Such situation occurs in 1 - 4% of patients with transitional cell carcinoma [14]. It seems that the diagnosis of carcinoma in situ or multifocal carcinoma is a prognostic factor of infiltration the prostate by bladder cancer [15]. Why is the diagnosis of prostate infiltration by urothelial cancer so essential? The answer to the question lies in the analysis of cancer-specific survival (see figure 1 and proportional hazard regression), though some authors emphasize the fact that the infiltration of excretory ducts by urothelial cancer is not an unfavorable phenomenon in comparison to the infiltration of the prostatic stroma [16]. It seems that the best way to evaluate the infiltration of prostrate by urothelial cancer before operation is to use diagnostic cystoscopy. The risk of failure to diagnose the infiltration in that type of examination is really low, equal to 3.1% [15]. In our centre each patient undergoes cystoscopy before the operation of bladder removal. In our study 50 patients (96,1%) had positive cystoscopy in terms of prostate infiltration prior to bladder removal. The range of urethra's infiltration by urothelial cancer also influences the choice of urinary diversion after cystoprostatectomy and possibly carrying out excision of urethra.

Another problem seems to be the presence of prostatic adenocarcinoma. Referring to the results of the following research, it can be stated that patients with bladder cancer stage pT_4 _have significantly worse distant results than patients with co-existing prostate cancer. The reason for that situation is certainly the fact that prostate cancer in most cases was well differentiated, with low stage of pathologic advancement. The frequency of prostate cancer co-existence in the specimen after cystoprostatectomy is approximately 30% - 40%, out of which only 20% are clinically significant [17-20].

Patients who have been diagnosed with the simultaneous existence of both prostate and bladder cancer require close monitoring, focusing on the possible relapse of prostate cancer after cystoprostatectomy. In the following research biochemical recurrence occurred in 2 (9%) patients after radical cystoprostatectomy. Urological literature indicates that the number of relapses in similar cases can be significantly higher, up to 40%, and death caused by relapses occurs in 17.6% of patients during 10 years of observation [21].

Due to both aforementioned situations, the risk of non-radicality of prostate sparing cystectomy is relatively high. Both the co-existence of prostate cancer and the infiltration of prostate by urothelial cancer in patients with infiltrating bladder cancer is a negative prognostic factor. The abovementioned situations confirm that the treatment of choice in case of infiltrating bladder cancer is radical cystoprostatectomy.

## Conclusions

1. Incidentally diagnosed prostate cancer in specimen after cystoprostatectomies carried out due to muscle-invasive bladder cancer is frequently clinically insignificant and characterized by low progression.

2. The risk of simultaneous prostate cancer in patients with bladder cancer is so high that performing prostate sparing cystectomy requires the broadening of preoperative diagnostics and special indications.

3. Patients with bladder cancer infiltrating prostate are characterized by much higher percentage of progression and death in comparison with patients with co-existing prostate cancer.

## Competing interests

The authors declare that they have no competing interests.

## Authors' contributions

PB prepared a study concept and design as well as made statistical analysis. MF and GP analysed and interpreted all data. KP and PP acquired all data. ZK drafted the manuscript. AP and MF were the supervisors. All authors read and approved the final manuscript.

## Pre-publication history

The pre-publication history for this paper can be accessed here:

http://www.biomedcentral.com/1471-2490/10/16/prepub
